# Nanostructured titanium surfaces exhibit recalcitrance towards *Staphylococcus epidermidis* biofilm formation

**DOI:** 10.1038/s41598-018-19484-x

**Published:** 2018-01-18

**Authors:** Yunyi Cao, Bo Su, Subash Chinnaraj, Saikat Jana, Leon Bowen, Sam Charlton, Pengfei Duan, Nicholas S. Jakubovics, Jinju Chen

**Affiliations:** 1 School of Engineering, Newcastle University, Newcastle Upon Tyne, NE1 7RU UK; 2 School of Oral and Dental Sciences, University of Bristol, Bristol, BS1 2LY UK; 3 Department of Physics, Durham University, Durham, DH1 3LE UK; 4 School of Dental Sciences, Newcastle University, Newcastle Upon Tyne, NE2 4BW UK

## Abstract

Titanium-based implants are ubiquitous in the healthcare industries and often suffer from bacterial attachment which results in infections. An innovative method of reducing bacterial growth is to employ nanostructures on implant materials that cause contact-dependent cell death by mechanical rupture of bacterial cell membranes. To achieve this, we synthesized nanostructures with different architectures on titanium surfaces using hydrothermal treatment processes and then examined the growth of *Staphylococcus epidermidis* on these surfaces. The structure obtained after a two-hour hydrothermal treatment (referred to as spear-type) showed the least bacterial attachment at short times but over a period of 6 days tended to support the formation of thick biofilms. By contrast, the structure obtained after a three-hour hydrothermal treatment (referred to as pocket-type) was found to delay biofilm formation up to 6 days and killed 47% of the initially attached bacteria by penetrating or compressing the bacteria in between the network of intertwined nano-spears. The results point to the efficacy of pocket-type nanostructure in increasing the killing rate of individual bacteria and potentially delaying longer-term biofilm formation.

## Introduction

Titanium (Ti) and its alloys have been widely used for biomedical devices and surgical implants, due to their excellent mechanical properties, corrosion resistance and biocompatibility^1–3^. However, the performances of these Ti-based implants are adversely affected by bacterial growth that can cause infections and inflammation of the surrounding tissues^4–6^. The primary cause of prosthetic implant infections (PIIs) is pre-surgical contamination following bacterial adhesion^7,8^, which can cause acute infections between 6 days to 6 weeks after placing the implant^9^. Chronic PIIs are usually associated with the formation of bacterial biofilms^7^. Bacterial cells can colonize the surfaces of implants and become embedded within a self-secreted matrix of extracellular polymeric substances (EPS)^10–12^, which is a prelude to infection and consequently to tissue destruction, systemic dissemination of the pathogen^13^. The biofilm protects the encased bacteria from the host immune system and from external antimicrobial agents, thereby raising the instances of infections amongst patients^14–16^. And the resistant PIIs can only be solved by surgical removal of implants^7^. According to the U.S. National Institutes of Health (NIH), up to 80% of human bacterial infections are caused by biofilms and with more than 2 million annual cases, resulting in $5 billion losses in the U.S. alone, it is clear that biofilms cause an extreme burden on health services^17,18^. Some bactericidal implants have been manufactured by coating with antimicrobial agents such as antibiotics (e.g. tobramycin), oxidants (e.g. chlorine), or biocides (e.g. silver nanoparticles) on the implant surfaces to kill bacterial cells^19^. However, this strategy is not efficient for long-term implants such as those used in hip or knee replacements. Over long times, the concentration of the antimicrobial agent depletes which can result in the development of antimicrobial resistance^7^, as has been shown for *Staphylococcus epidermidis*^20,21^ for example.

To circumvent the problem of antimicrobial resistance, physical methods involving surface topography modification with micro- and nanostructures have shown potential for inhibiting adhesion or killing microbial cells that contact the surface. For example, lotus leaf^22–24^ and shark skin^25,26^ exhibit anti-fouling properties because of their special surface topography and have inspired the development of various biomimetic materials. Lotus-inspired superhydrophobic TiO_2_ nanotube arrays were able to inhibit the adherence of *Staphylococcus aureus* due to a self-cleaning effect^22^ wherein the water droplets roll off the patterned surface, thereby sweeping away dust/dirt and microorganisms^27,28^. Micro-scale patterns mimicking sharkskin on poly dimethylsiloxane (PDMS) polymer has led to the development of Sharklet AF^TM^, which has shown effectiveness in blocking biofilm development by acting as a physical obstacle to colonization^25^. However, such patterns are very difficult to transfer to stiff materials like titanium.

Other contemporary approaches for inhibition of microbial adhesion or contact killing have drawn inspiration from the surface topography of gecko skin^29,30^, wings of dragonfly^31,32^ and cicada^28,33^. The naturally occurring high aspect ratio nanopillar-structures on these surfaces can potentially pierce cell membranes and cause bacterial death. Previous studies have artificially reproduced nanostructures on titanium surfaces with features akin to the wings of dragonfly^34^ or cicada^35^ using hydrothermal treatment methods. For example, Bhadra *et al*.^34^ showed that dragonfly-inspired TiO_2_ nanowires aided in bactericidal activity against Gram-negative *Pseudomonas aeruginosa* and Gram-positive *Staphylococcus aureus* cells^34^. This surface also promoted the attachment of primary human fibroblasts. Our prior work^35^ has demonstrated that very similar TiO_2_ nanostructures grown on Ti were not only capable of regulating mammalian cell proliferation, but also were bactericidal against several bacterial species including *Pseudomonas aeruginosa*, *Escherichia coli* and *Bacillus subtilis*. Ultimately, contact-killing approaches may be further improved by the inclusion of antimicrobial release systems to promote killing at a distance. However, first it is necessary to elucidate the mechanisms underlying the physical contact-based bactericidal activity, since these are not clear at present. In addition, there is a lack of knowledge about how nanostructures with different architectures affect bacterial growth over a longer period of time.

In this paper, we studied short-term bacterial adhesion and longer-term biofilm formation on two different architectures of nanostructured titanium surfaces synthesized using the hydrothermal treatment method as described in our prior work^35^. A two-hour treatment (i.e. spear-type) generated short and thin nano-spears which bear resemblance to cicada wings. While a three-hour treatment (i.e. pocket-type) generated an open porous nanostructure, which consisted of intertwined nano-spears. A clinically relevant bacterium *Staphylococcus epidermidis* (i.e. *S. epidermidis*) was grown on the nanostructured and/or polished titanium surfaces; and its interaction with the surfaces were visualized using the combination of Confocal Laser Scanning Microscopy (CLSM) and Focused-ion-beam Scanning Electron Microscopy (FIB-SEM). The results indicated that the pocket-type nanostructure can delay biofilm formation up to at least 6 days by causing mechanical rupture or severe deformation of bacterial cell membranes. We anticipate that these insights will facilitate the development of novel bactericidal nanostructured implant surfaces that reduce biofilm growth thereby reducing the frequency of infections.

## Results and Discussion

### Characterization of nanostructures on titanium surfaces

**Figure 1:**
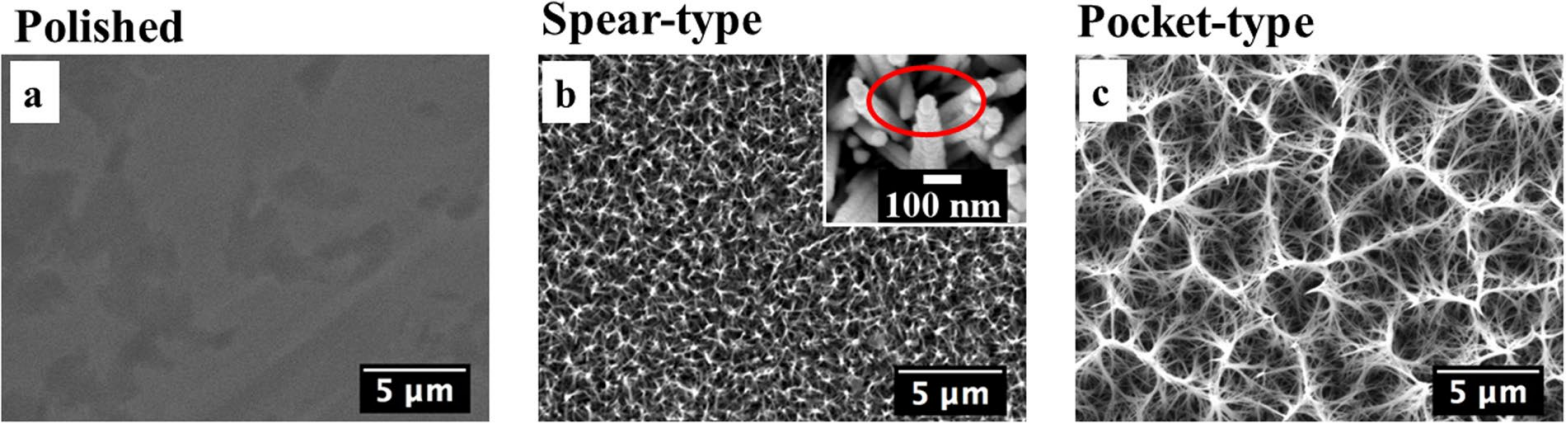
SEM images of different titanium surfaces at magnification of 8000×. (**a**) Polished titanium surface. (**b**) Spear-type surface showing a dense nanostructure comprising short and thin spears. The inset shows a SEM image of a nano-spear with a cone-shaped cap taken at 100,000×magnification. (**c**) Pocket-type surface formed by intertwining of the longer and wider nano-spears.

Initially the physical properties of different titanium surfaces were investigated. Figure 1a displays an SEM image of the polished titanium surface which had an average roughness (Ra) value of 13.2 ± 2.3 nm (Table 1). Hydrothermal treatments were performed on the polished disks and the duration of treatment determined the variability patterns on the surfaces. A two-hour hydrothermal treatment at 240 °C generated dense and periodic structures (Ra ~ 195.0 ± 6.5 nm, see Table 1) consisting of nano-spears with base diameter of approximately 70 nm, denoted as spear-type (Fig. 1b). The top of each nano-spear consisted of a cone-shaped cap (see magnified image Fig. 1b, upper right); the tip rounding of the cone was around 50 nm and the cone angle was found to be approximately 55°. The cone-shaped cap of the nano-spear was a result of crystal growth during hydrothermal treatment, and similar observations have been reported in the previous investigations^36,37^. Such structural features are similar to the conical nano-pillars found on cicada wings (periodic spherically capped and in the diameter of 60 nm^28^). On the other hand, a three-hour hydrothermal treatment generated an open porous nanostructure with clusters of pockets with diameter of 3–5 µm, and was denoted as pocket-type (Fig. 1c). These pockets were formed by the intertwining of relatively long, randomly oriented nano-spears with diameter of around 100 nm. The pocket-type structure resulted in a much rougher surface (Ra ~ 479.0 ± 15.3 nm, see Table 1) than either of the other surfaces used in this study. The maximum roughness depth (Rmax) of the spear and pocket-type surface reached up to 1,862.0 ± 121.0 nm and 4,098.0 ± 239.0 nm, respectively, indicating that the growth of nano-spears took place during the hydrothermal process which led to a much higher topography on the pocket-type titanium surface.

**Table 1:** Characterization of structures present on the different titanium surfaces. Ra represents the average roughness, Rms represents the root mean square roughness, and Rmax represents the maximum roughness depth, respectively. Surface area was the real surface area that AFM scanned which was under the same projected area of 100 µm^2^ in this study.

Surfaces	Ra (nm)	Rms (nm)	Rmax(nm)	Surface area (um^2^)	Spear size(nm)	Overview
*Polished*	13.2 ± 2.3	32.0 ± 4.2	968.0 ± 87.0	100.8	—	flat surface
*Spear-type*	195.0 ± 6.5	245.0 ± 7.6	1862.0 ± 121.0	200.5	70	short and thin nano-spears
*Pocket-type*	479.0 ± 15.3	619.0 ± 12.5	4098.0 ± 239.0	1425.8	100	clusters of pockets in the diameter of about 3–5 µm

X-ray photoelectron spectroscopy (XPS) analysis revealed the existence of Carbon (C), Oxygen (O) and Titanium (Ti) in each of the surfaces as shown in Table S1. The XPS spectrum of the Ti 2p peak (See Figure S1) also confirmed the formation of titanium dioxide (TiO_2_) on both spear and pocket-type surfaces. Surface wettability was assessed through contact angle measurements using deionized (DI) water, diiodomethane and glycerol (values shown in Table S2). Both spear and pocket-type surfaces exhibited superhydrophilicity during measurement with DI water/diiodomethane and the droplets of the two liquids tended to spread on the surface within 1–3 seconds. For glycerol, droplets remained sessile for the first 5 seconds during the measurement, but spread thereafter. The superhydrophilicity of the nanostructured titanium surfaces was a result of the heat-treatment, which removed the adventitious hydrophobic organic surface species^38^. Another reason was the dissociative adsorption of water at the surface defects owing to the existence of Ti^2+^ or Ti^3+^ (see Figure S1), which caused the oxygen vacancies and was expected to increase the adsorbed hydroxyl group density thereby forming hydrophilic regions^38^.

### Visualization of the initial bacterial attachment and interaction with surfaces

**Figure 2:**
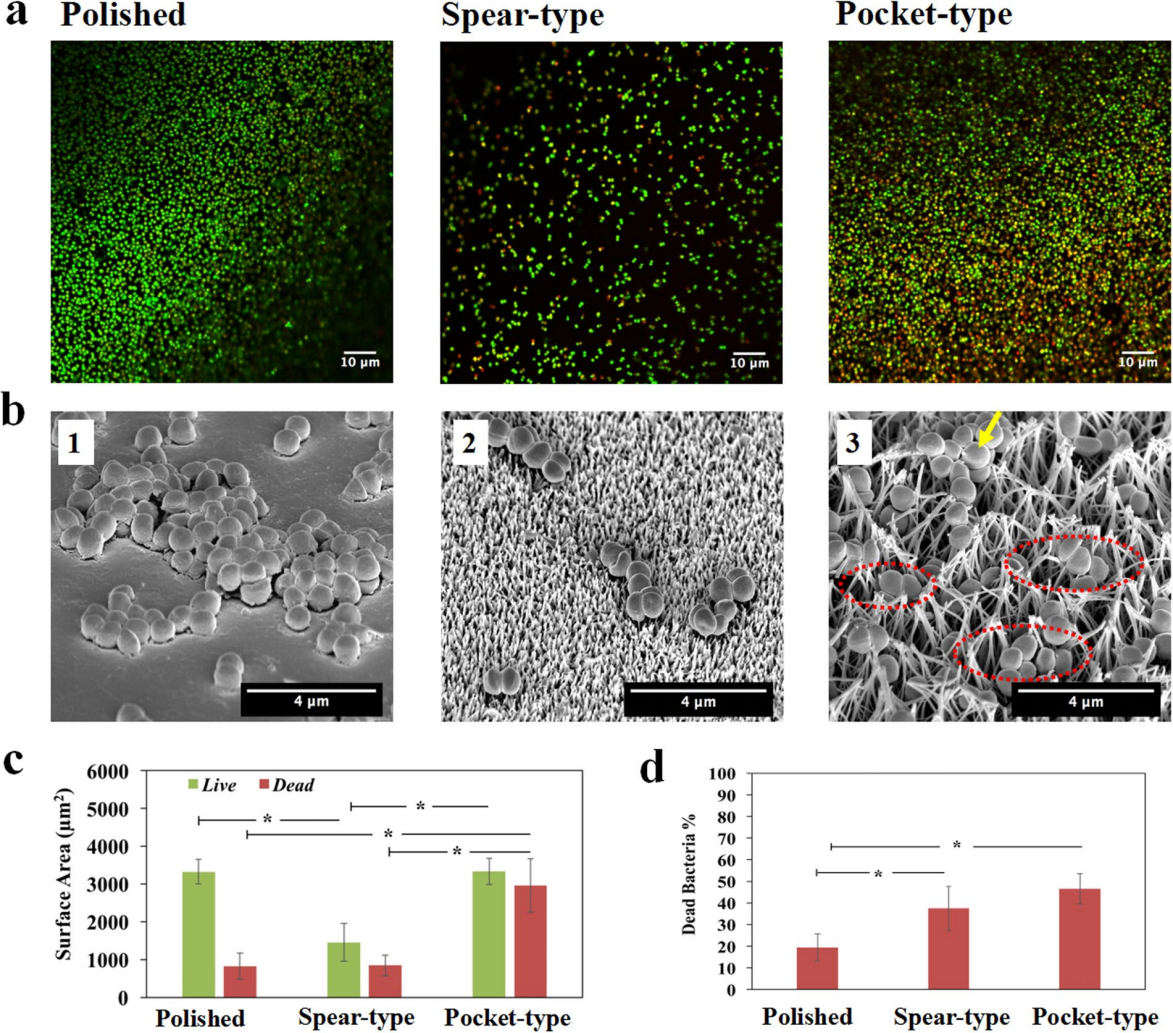
Adherence of *S. epidermidis* on different titanium surfaces after 2 hours’ incubation. (**a**) Representative CLSM images with LIVE/DEAD staining of *S. epidermidis* on polished, spear-type and pocket-type titanium surfaces (left to right). (**b**) Different contact mechanisms of *S. epidermidis* on different titanium surfaces as visualized by SEM (Images were taken at 40° tilt with magnification of 25000×). (b1) *S. epidermidis* cells rested on the polished titanium surfaces; (b2) for spear-type surfaces, *S. epidermidis* cells settled on the top of spears resulting in point contacts; (b3) for pocket-type surfaces, *S. epidermidis* settled inside the pocket-like nanostructures (outlines of selected pockets are marked by dashed red lines). The longer, intertwined nano-spears were also observed to provide colonization sites for bacteria (arrow). (**c**) Surface area covered by both live and dead bacteria in the field of view for each titanium surface. (**d**) Bactericidal efficiency of the different titanium surfaces, as indicated by the proportions of dead cells among the adherent bacteria. *Statistically significant difference (*p* < 0.05). Three independent experiments were performed for each substrate type.

After the bacteria in the culture medium encounter the surface, they can strongly adhere onto the surface as a key initial step in the formation of biofilms. We assessed the adherence of *S. epidermidis* to the different surfaces after two hours’ incubation by performing live/dead visualization with CLSM (see Fig. 2a) in which live bacterial cells were stained green with SYTO^®^9 and dead cells were stained red with propidium iodide. The total attachment (of live and dead cells) on titanium surfaces was found to be ranked in the order: pocket-type > polished > spear-type as seen from Fig. 2c. Of the three surface types, the pocket-type surface harboured the largest surface area covered by bacteria. Both polished and pocket-type surfaces had a similar coverage of live bacteria in the field of view (*p* = 0.96); however, the surface coverage of live bacteria on the spear-type nanostructure was only half of that on the polished titanium. Next we sought to quantify the bactericidal activity of the surfaces. A standard approach for counting live bacterial cells is to remove them from the surface and enumerate Colony Forming Units (CFU) on an appropriate solidified growth medium. However, nanopillar-structures can promote strong adhesion of cells to surfaces, making it difficult to recover viable cells without damaging them^34,35^. Even on unmodified surfaces, it is difficult to recover all cells and to break up attached aggregates into single cells without killing them^39,40^. Therefore, the bactericidal efficiency of titanium surfaces (Fig. 2d) was determined by directly assessing the proportion of dead (red) cells from CLSM images using the BacLight cell viability kit, which is a widely used method^7,34–36,41,42^. From this, the percentage of dead cells on these titanium surfaces was ranked as follows: pocket-type (47%) > spear-type (37%) > polished surface (19%). Overall, the nanostructured titanium surfaces exhibited higher bactericidal efficiencies than the control, which is in agreement with previous studies^34,35,41^.

To understand the interactions between the cell and surfaces, we used SEM to visualize the interaction of *S. epidermidis* with different surface structures. Since the measured diameter of *S. epidermidis* was 0.71 ± 0.07 µm in this study and the polished surface had the least roughness with average roughness value (Ra ~ 13.2 ± 2.3 nm, see Table 1), the cells were well attached onto the smooth surface and had a large contact area (Fig. 2b1). For the spear-type, the inter-spear distance was measured to be 191.09 ± 120 nm (Figure S2), which was much smaller than bacterial cells. The periodic pattern of the structure resulted in reduced contact area between bacteria and the nano-spears, thereby leading to fewer colonization hotspots (~3–6 point contact regions based on the nominal diameter of cell) when compared with the polished surface (Fig. 2b2). The pocket-type nanostructure comprised of a three-dimensional porous structure, which led to the largest surface area under the same projected area as measured by AFM (Table 1). For the pocket-type, single bacteria can settle inside/around the top rim of pockets or slide in between tilted spears, thereby increasing the number of contacts around the cell (Fig. 2b3). This contrasted with the spear-type surface where the point contacts only happened at the bottom of the cell. The distribution of contacts all around the cell body in the pocket-type structure can possibly explain the larger fraction of attached cells. Collectively, the total surface areas that can be in contact with bacteria in this study were ranked as: pocket-type > polished > spear-type, which agreed with the experimental results as shown in Fig. 2c. Therefore, we hypothesized that the topographical patterns played a crucial role in the bacterial interaction with surfaces and determine their fate on the different kinds of surfaces.

### Prediction of bacterial attachment on different surfaces using XDLVO theory

**Figure 3:**
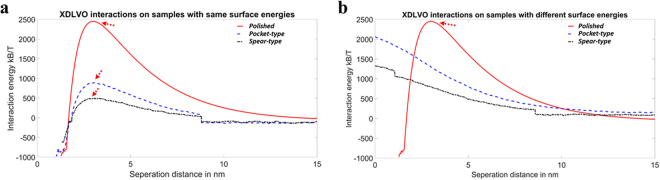
(**a**) Interaction energies for the three titanium surfaces using the different surface roughness values as measured on each individual sample, but using the same surface energy parameters as measured on the polished one. The red arrows indicated the repulsive maximum barrier. (**b**) Interaction energies for the three titanium surfaces using different surface roughness values and different surface energy parameters as measured on each individual sample, respectively. The red arrows indicated the repulsive maximum barrier.

The attachment of bacteria on surfaces is affected by the roughness, hydrophobicity, electrostatic double layer and various other factors^7,19,43–45^. To understand the effects of the various components on the bacterial attachment onto surfaces, we performed simulations using the extended Derjaguin–Landau–Verwey–Overbeek (XDLVO) theory to calculate the interaction energy between bacteria and surface. We initially evaluated the effects of surface roughness on bacterial adhesion with the same hydrophobicity (Fig. 3a). Bacteria need to overcome the energy barriers to reach the negative energy regions thereby facilitating the bacterial attachment. It was evident that the energy barrier was the lowest for spear-type and highest for the polished one (Fig. 3a). A higher energy barrier leads to a lower bacterial attachment and thus the simulation (Fig. 3a) indicated that the ranking of bacterial attachment was: spear-type > pocket-type > polished, which did not agree with the experimental results shown in Fig. 2c. This may indicate that surface roughness is not the dominant factor for bacterial attachment in this study. It has been reported that bacterial attachment may be enhanced when the roughness is above a certain threshold (about 200 nm)^44,46^. However, Lorenzetti *et al*.^7^ reported that for titanium-based substrates with roughness between 300–800 nm, bacterial attachment was reduced with the increase of roughness. Bacterial attachment strongly depends on the surface topography instead of roughness at a nanoscale^7,47^, which not only affects the cell-material interactions, but also changes the surface wettability^45,47–50^. Since the simulation above did not account for the different hydrophobicity of surfaces, another simulation was performed by considering different roughness and the resultant surface hydrophobicity differences (Fig. 3b). The simulation revealed that the spear-type and pocket-type surfaces would repel the bacteria as both interaction energies were positive even approaching the surfaces (see Fig. 3b). For the polished one, it can overcome the energy barrier to reach the region where the interaction energy was negative to make the bacterial attachment happen. Then the ranking of bacterial attachment was: polished ≫  spear-type > pocket-type. This ranking still did not agree with the experimental observation shown in Fig. 2c, which indicated that hydrophobicity of the architectured surfaces of material itself was also not a key factor for bacterial attachment under the conditions used in this study. A possible mechanism is that the nanostructured titanium surfaces can adsorb the proteins in the growth medium^49–51^. This pre-adsorbed protein layer may alter the surface hydrophobicity prior to bacteria arrival but may not significantly affect the surface structure^50^. While at a nanoscale, surface topography rather than surface roughness would be the dominant factor in this study, and the accessibility of bacteria to the contact surface is the decisive factor. Roughness only reflects unevenness in height (z) direction. The current study shows that lateral dimensions in x, y directions (i.e. diameter of nano-spears and spacing between nano-spears) are far more important parameters. Surface roughness is no longer a valid indicator and surface architecture plays the dominant role in bacterial attachment in this study as predicted.

### Cell membrane damage by nanostructures leads to bacterial death

**Figure 4:**
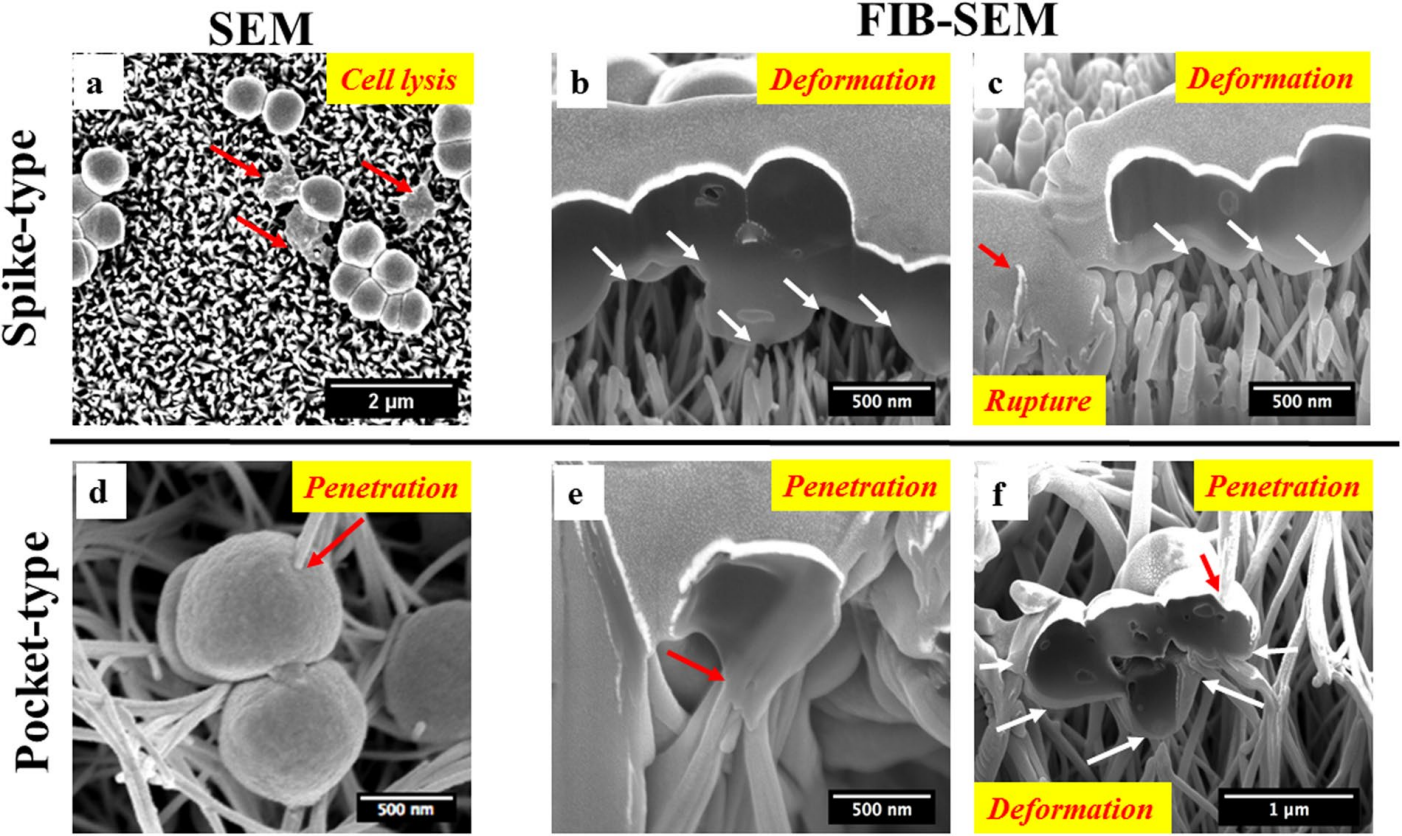
Bactericidal activities of the spear-type and pocket-type surfaces after 2 hours’ incubation. (**a**) SEM images showing morphology of *S. epidermidis* on the spear-type surface. Some bacteria were flattened indicating cell death (red arrows). (**b**,**c**) The interaction between *S. epidermidis* cell membrane and the nanostructured spear-type surface, visualized by FIB-SEM. The sharp tips of nano-spears on surfaces resulted in the deformation of bacteria cell membranes (white arrows). The red arrows represent cell rupture and leakage of cytoplasm, which extended down into the nanostructure. (**d**) Representative SEM images showing *S. epidermidis* cell membrane directly penetrated by the longer nano-spears inside the pockets (red arrow). (**e**,**f**): The interaction between *S. epidermidis* cell membrane and the nano-spears of pocket-type, as visualized by FIB-SEM. The bactericidal activities of these nanostructures were a result of the combined effect of the penetration (red arrows) and the stretching/compression from the nano-spears (white arrows).

The higher fraction of dead bacteria on nanostructured titanium surfaces (Fig. 2d) may be a result of mechanical interactions between bacteria and the nanostructures. The possible reason is that physical contact has been shown to cause membrane deformation and puncture on other types of artificially synthesized surfaces^34,35^. To investigate the mechanical mechanisms responsible for cell death in this study, we undertook FIB-SEM for *S. epidermidis* growing on the different kinds of surfaces. On the spear-type titanium surface, a fraction of the attached *S. epidermidis* (Fig. 4a) showed a flattened cell shape. The bacteria making contacts with nano-spears can be severely deformed/penetrated by the sharp tips (tip radius ~50 nm) resulting in a leakage of cytoplasm (Fig. 4b,c) and subsequent cell lysis^52,53^. For the pocket-type structure, the bacteria were able to fall into the pockets due to a combination of gravitational and/or surface adhesive forces^30^. This can result in the penetration of cell membranes by the randomly oriented nano-spears inside the pockets (Fig. 4d,e). All these bactericidal activities happened on both surfaces when the adhesive force between bacteria and nanostructures overcomes the membrane tension force that initially keeps the cell in shape^28,30,35^. For pocket-type structure, the bacteria can also be squeezed between the nano-spears as shown in Fig. 4f. The forces acting on the bacteria membrane arising from the sidewalls of nano-spears can cause severe stretching/compression of the cell membranes (Fig. 4f). This additional bactericidal mechanism of the pocket-type structure is likely to be responsible for the higher antimicrobial efficiency as compared with the spear-type.

In this study, we aimed to investigate the physical contacted-based bactericidal mechanism of nanostructured titanium surfaces. The goal of this approach is to inhibit bacterial adhesion or to kill bacterial cells that contact the surface. The advantage of this approach over chemical release-based bactericidal surfaces is that it is unlikely to promote antibiotic resistance^54^. However, a potential drawback is that surface modification alone cannot kill cells at a distance. Indeed, we observed strong growth of bacteria in the culture medium above the surfaces when culturing biofilms, indicating that there was no killing at a distance. In fact, it is important that the nanostructures are not released since this could potentially lead to toxicity to the host^55^. Ultimately, the development of bactericidal nanostructured surfaces may pave the way for the development of a new generation of biomaterials that use a combination of physical contact-based killing and chemical release-based methods to kill bacterial cells at a distance without inducing toxicity to the host. In this regard, the pocket-type titanium surface has shown the greatest potential as an optimised structure which can physically kill the attached cells by contact. Next, we aimed to investigate that if this pocket-type surface can exhibit anti-biofilm effects over a longer timeframe.

### Two-day and Six-day biofilm growth is delayed by the pocket-type nanostructure

#### Biofilm growth for two days on different surfaces

**Figure 5:**
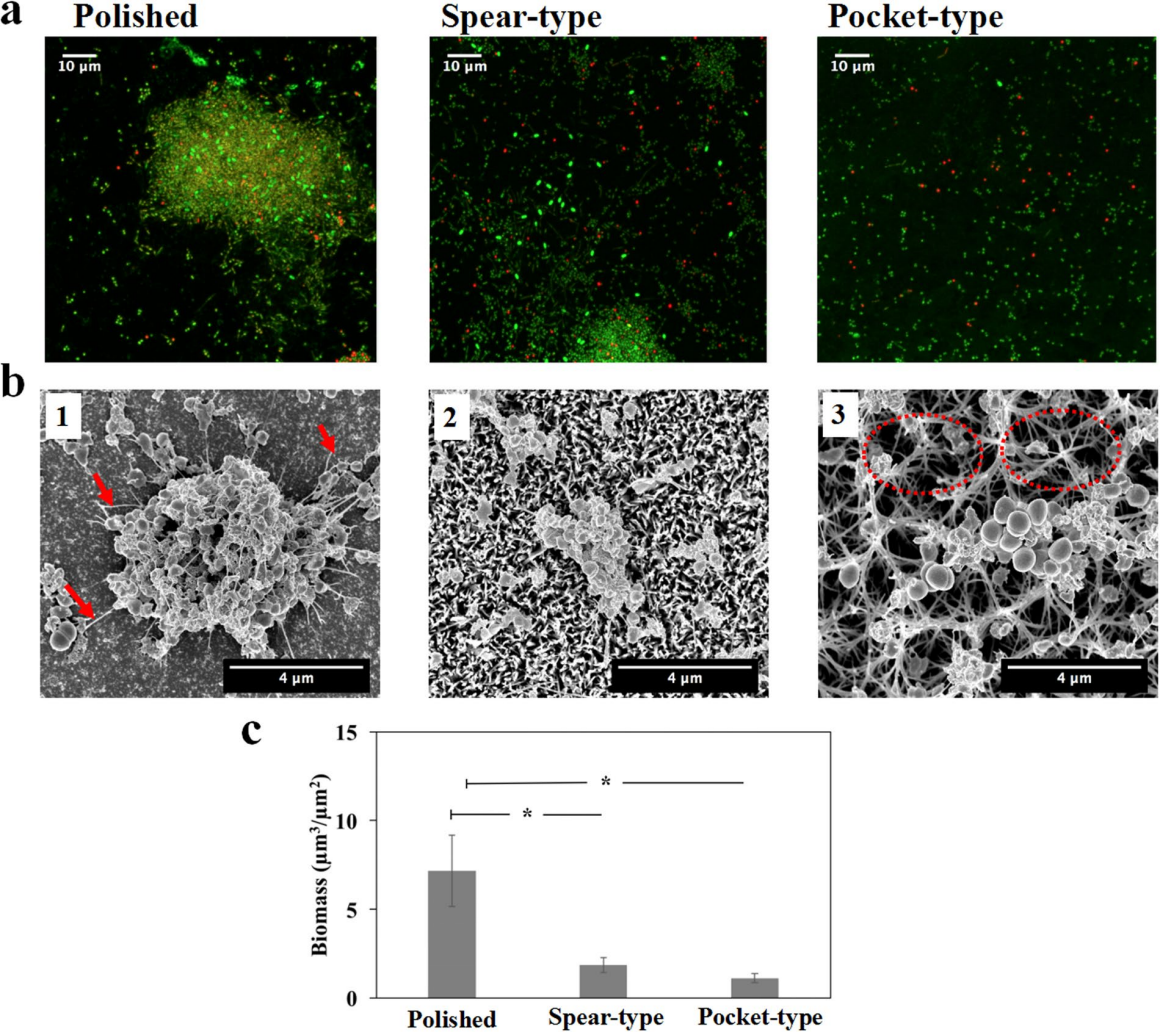
Early stage *S. epidermidis* biofilms grown on different surfaces over a period of 2 days. (**a**) Representative CLSM images with LIVE/DEAD staining on three different surfaces, shown as maximum intensity projections through the thickness of the biofilms. (**b**) SEM images of *S. epidermidis* biofilm visualized at magnification of 25000×. (b1) The polished titanium surface displayed dense bacterial clusters, with slime (EPS) lining the cell clusters (red arrows) (b2) Small clusters of bacteria are seen on the spear type surface. (b3) The pocket-type surface shows no significant bacterial clusters inside the pockets (red dash lines). (**c**) Biomass volume per area on the nanostructured substrates. *Statistically significant difference (*p* < 0.05). Three independent experiments were performed for each substrate type.

To determine whether nanostructures on titanium surfaces are effective in delaying the growth of biofilm networks, S. *epidermidis* biofilm was grown for 2 days and then visualized using CLSM and SEM (Fig. 5a,b). The total biomass on the polished surface was found to be almost 4 times more than that on spear-type surfaces and about 6 times more than that on pocket-type surfaces (see Fig. 5c). The polished titanium surfaces harboured *S. epidermidis* biofilm clusters with well-connected EPS as shown in the SEM image (Fig. 5b1). A few smaller clusters were found on the spear-type structure (Fig. 5b2) and only very small aggregates comprising ~10 cells were observed on the pocket-type structure (Fig. 5b3). This indicated that *S. epidermidis* biofilms showed the least growth on the pocket-type titanium surfaces at the end of two days.

### Biofilm growth for six days on different surfaces

**Figure 6:**
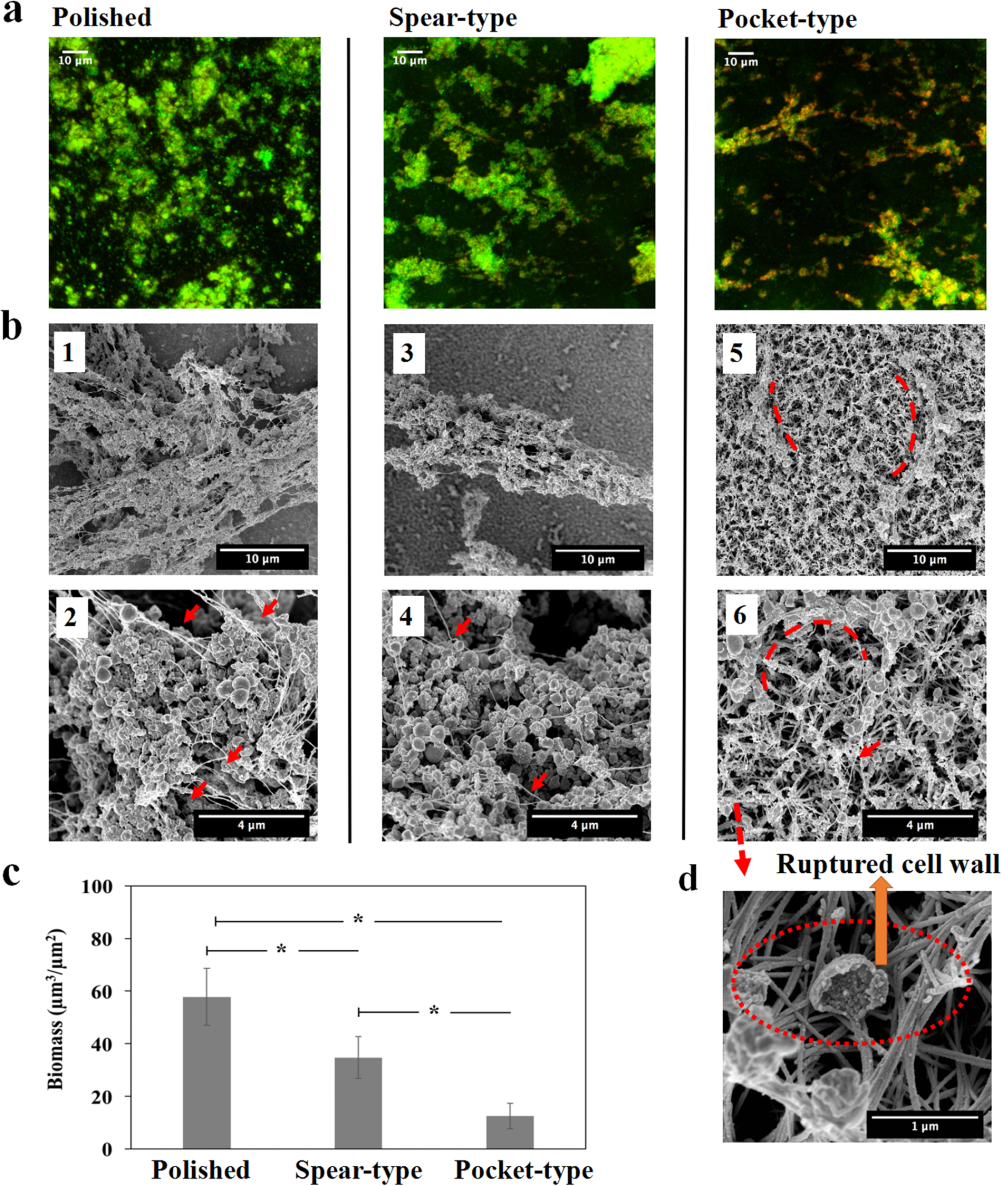
*S. epidermidis* biofilms grown on different surfaces over a period of 6 days. (**a**) Representative CLSM images with LIVE/DEAD staining on different surfaces. (**b**) The images (1, 3, and 5) were taken at magnification of 8000×; Images (2, 4, and 6) were higher magnification (25000×) of the biofilms. More mature biofilms were found on the polished and the spear-type titanium surfaces, with extensive EPS (red arrows). By contrast, the pocket-type surface continued to exhibit small bacterial clusters that tended to wrap (red dash lines) around at the nano-spears that formed the pockets, with relatively little EPS (red arrows). (**c**) Biomass volume per area on the nanostructured substrates. *Statistically significant difference (*p* < 0.05). (**d**) SEM image taken at magnification of 100, 000×, showed that S*. epidermidis* cells were further damaged and collapsed inside the pockets (red dash line) after 6 days. Three independent experiments were performed for each substrate type.

We also studied biofilm growth for a period of 6 days which is longer than the duration that has been studied in the existing literature for anti-biofilm evaluation, since the existing micro or nanostructured antibacterial surfaces only demonstrated the capability to delay the biofilm formation by up to 2 or 3 days^48,56,57^. The maximum intensity projections through the thickness of biofilms and the SEM images for the different surfaces are shown in Fig. 6a,b. The total biomass on the polished surface was found to be almost 2 times more than that on spear-type surfaces and about 5 times more than that on pocket-type surfaces (see Fig. 6c). Figures 6b and (1–2) showed the existence of a dense biofilm network on the polished titanium surface. The size of the cluster was significantly larger than those seen at the end of day two (Fig. 5b1). The spear-type surface also showed an increase in the size of biofilm clusters, and the existence of EPS was observed as the presence of string-like structures that were seen in Figs 6b and (3–4). Figures 6b and (5–6) showed that only small bacterial clusters were found on the pocket-structure with the little EPS lining the topmost rims. On the other hand, the SEM image with 40° tilt (Figs 6d and S3) indicated that bacteria cells were further damaged and collapsed inside the pockets, which confirmed the absence of any substantial bacterial clusters inside the pore spaces of the 3D network. Thus, we concluded that the pocket-type titanium surface can significantly inhibit biofilm formation over a period of 6 days.

### Proposed anti-biofilm mechanisms on different surfaces

**Figure 7:**
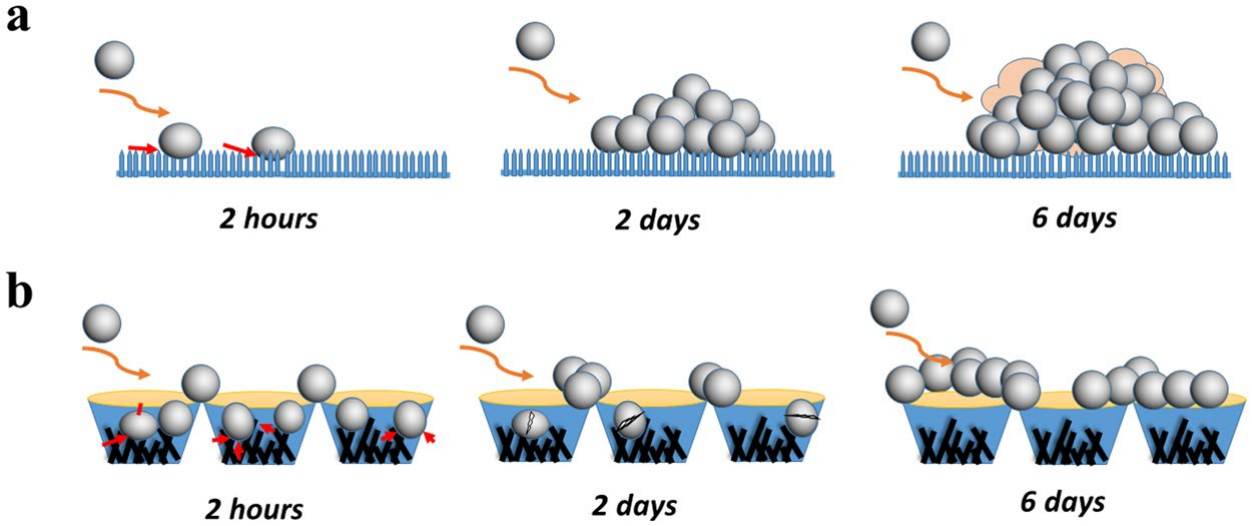
Hypothesized anti-biofilm mechanisms for the transition from 2-hour to a 6-day biofilm on nanostructured titanium surfaces. (**a**) For spear-type, few bacteria are initially attached (i.e. at end of 2 hours) due to a lower adhesive strength caused by the point contacts between bacteria and surface. The attached cells develop small bacterial clusters and subsequently form thick biofilms after 6 days. (**b**) For pocket-type, more bacterial cells compared to spear-type are initially attached. Bacterial cells inside the pockets become immobilized/isolated and some are penetrated/stretched by the nano-spears inside the pockets. However, cells can aggregate at the top rim of the pocket to form bacterial clusters after 6 days.

The dynamics of growth and subsequent biofilm formation on different surfaces based on the different imaging techniques can be described according to the schematic in Fig. 7. For the spear-type titanium surface, the spacing between the spears is smaller than the size of bacterial cells and they are periodically arranged. Therefore the bacteria are only able to position themselves at the tips of the nano-spears. A few bacteria are initially attached on the surface due to the lower contact area and they can grow freely in the direction normal to the surface. Since the distance between the spears is very small, the packing pressure generated due to bacteria growth cannot push the cells into the voids of the spear-type surface (Fig. 7a). As a result, bactericidal activity is not efficient. It is possible that the accumulation of dead bacteria/ debris can also serve as a conditioning film to provide nutrients and adhesion receptors for subsequent bacterial attachment^58^. Thus, a thick biofilm is formed over a period of 6 days on the spear-type surface.

The pocket-type structure which has an open porous network of randomly oriented spears presents an interesting situation. Due to the voids in the network, bacteria can either get lodged inside the pocket or settle on the top rim. The total number of initially attached bacteria is usually much higher than on the spear-type surface. If the cells are trapped inside the pockets, they can be killed either by direct membrane penetration or severe membrane deformation. The top rim of the pocket is relatively blunt compared to the nano-spears inside the pocket, which reduces the possibility of puncturing the cells. Therefore, a fraction of the cells deposit on the rims and can multiply over the time. As the cells grow and form a layer of biofilm, the build-up of the pressure in the network can cause the layers of cells to be pushed into the voids resulting in cell death by direct penetration/compression from the spears. The dead cells decay over time leaving little bacterial clusters inside the voids of the pocket-type structure as indicated by Figs 6d and S3. As a result, we can only see bacteria on the top rim of the pockets, which tended to aggregate and form small bacterial clusters (Fig. 7b). Thus this pocket-type surface shows efficacy in delaying biofilm formation for up to 6 days.

Most studies on bactericidal nanostructured surfaces have focussed on the early stages of bacterial biofilm formation^28,30,34,35,48,52,55,56,59,60^. There is a lack of investigations into longer-period biofilm growth. Here, this study described possible mechanisms for biofilm development on bactericidal nanostructured titanium surfaces up to 6 days, as shown here for the first time. We also studied biofilm growth over a period of 9 days (Figure S4). At this point, the pocket-type structure still exhibited anti-biofilm effects, which agreed with our hypothesis as shown in Fig. 7. These experiments were performed using a high bacterial concentration and incubating under high nutrient conditions optimised to promote bacterial growth. In the *in vivo* environment, the microbial challenge will usually be with a lower bacterial density and in the presence of many anti-bacterial host factors. Therefore, the inhibition of biofilm formation shown *in vitro* for the pocket-type titanium surface has shown promise for the development of a new anti-biofilm material, which thus can potentially inhibit acute implant infections occurring after 6 days or more^9^ following insertion of a device into the body. The ability of pocket-type nanostructure to inhibit colonisation may also help to combat late infections that occur by haematogenous spread. However, an important question is whether these nanostructured surfaces are mechanically stable *in vivo* and free from toxicity to the host. It has been demonstrated that these crystal-growth TiO_2_ nano-spears can support the growth and proliferation of human osteoblast-like cells (MG-63)^35^. It has also been argued that toxicity effects of nanostructured surfaces are related to the concentration of detached nanoparticles^55^ and thus further mechanical tests would be useful to examine if these nanostructures may be broken during the surgical operations. Nevertheless, this study paved the way for further development of sustainable anti-biofilm biomaterial surfaces to inhibit biofilm-associated implant infections.

## Conclusions

In summary, this study has comprehensively elucidated the mechanisms for bacterial attachment, bactericidal and anti-biofilm performances of different nanostructured TiO_2_ grown on titanium, against the Gram-positive biofilm-forming opportunistic bacterial pathogen *S. epidermidis*. We have demonstrated that the spear-type nanostructure was much more effective in terms of eliminating bacterial adhesion compared with the pocket-type nanostructure. Regarding the bactericidal efficiency, the spear-type nanostructure was slightly worse than the pocket-type. Even with much less amount of living bacteria initially attracted to its surface, the spear-type nanostructure exhibited unsustainable anti-biofilm effects because the dead bacteria can shadow the nano-spears to prevent killing of new arrivals. In contrast, the pocket-type nanostructure enabled sustainable anti-biofilm effects for at least 6 days in the vitro assay. Therefore, the pocket-type architecture can potentially be a very promising design for sustainable anti-biofilm nanostructured biomaterials. Further experiments using different bacterial species are required to test the effectiveness of the nanostructured surfaces developed in this study. In addition, mechanical tests and *in vivo* antibacterial tests in appropriate animal models (e.g. mouse or rat subcutaneous infection models) will also be conducted in the future.

## Materials and Methods

### Fabrication of Nanostructures on Titanium Surfaces

Hydrothermal treatments were performed on titanium disks as previously reported^35,41^. Disks of 12 mm diameter were cut from a chemically pure titanium sheet (Titanium Metals Ltd) and polished to a mirror shine (TegraPol-15, Struers). The disks were then sonicated in acetone for 10 minutes, air-dried and slotted into custom-made Polytetrafluoroethylene (PTFE) holders. Nanostructures were fabricated by immersing the titanium disks into a 125-mL acid-digestion vessel (Parr Instrument Company) containing 1 M NaOH (60 mL), and subsequently placed in an oven at a temperature of 240 °C for 2 and 3 hours, respectively. After the reaction, the vessels were then removed from the oven and allowed to cool to room temperature. Following this, the titanium disks were rinsed with deionized water (i.e. DI water) and subsequently heat-treated at 300 °C for 1 hour. To convert the hydrothermally synthesized sodium titanate into TiO_2_, the disks were immersed in 0.6 M HCl for 1 hour, then rinsed with water, and finally heat-treated at 600 °C for 2 hours.

### Characterization of Nanostructured Titanium Surfaces

The titanium surfaces were imaged by a JEOL JSM-5610LV scanning electron microscope (SEM), operated at 10 KV. Atomic Force Microscopy (i.e. AFM, Veeco Dimension 3100) was also performed to further characterize the surface topography with a silicon tip (radius~ 8 nm, MikroMasch), by scanning an area of 10 × 10 µm^2^ in tapping mode. AFM measurements were processed by Gwyddion^TM^ software to obtain surface roughness. For chemical analysis, a Thermo Scientific™ K-Alpha™^+^ X-ray Photoelectron Spectrometer (XPS) System was used to determine the compositions of the fabricated titanium surfaces. Peak fitting and data analysis were achieved with Casa XPS^TM^ software. Sessile drop contact angles (CA) on different titanium surfaces were also measured to characterize surface wettability. Three microliter droplets of deionized water (i.e. DI water), diiodomethane and glycerol were placed on the different titanium surfaces and their projections were analyzed using a CAM 100 optical contact angle meter (KSV Instruments Ltd., Finland). At least five droplet measurements were taken, and the results were presented as the mean contact angles with standard deviations.

### Bacteria culturing, attachment and biofilm growth protocols

*Staphylococcus epidermidis* FH8, isolated from a chronic rhinosinusitis patient at the Freeman Hospital, Newcastle Upon Tyne was used in this study^61^. *Staphylococcus epidermidis* FH8 was routinely cultured in Tryptic Soy Broth (TSB, Melford Laboratories Ltd, UK), in a shaker at 250 rpm, 37 °C for 16 hours and then diluted to OD_600_ = 0.30 with a spectrophotometer (Biochrom Libra S11, Biochrom Ltd., Cambridge, UK). Prior to seeding, the control (i.e. polished titanium) and the nanostructured titanium substrates were sterilized in a steam autoclave at 121 °C for 20 minutes, and added to wells of a 6-well culture plate.

To assay bacterial adhesion onto surfaces, 0.5 ml of the diluted bacterial culture was incubated with titanium substrates in 6-well culture plates for 2 hours at 37 °C and then removed for visualization. To examine the effect of nanostructures on growth and biofilm formation by surviving bacteria, we cultured the bacteria for up to 6 days. A large amount of bacterial suspensions was used to ensure that sufficient nutrients were provided and to avoid evaporation problems. 3 ml of bacterial suspension were added to each sample, and incubated for 2 days (early-stage biofilms) and 6 days (relatively mature biofilms). An incubation time of 2 days was chosen because previous reports indicated that the biofilm is not mature at 2 days, but still consists of bacterial cell clusters and microcolonies^21,48,56^. For the biofilm developed up to 6 days, half of the TSB medium was changed at day 3.

### Confocal Laser Scanning Microscope Analysis

The titanium substrates were removed from wells with sterile forceps and gently rinsed three times with Phosphate Buffered Saline (PBS, pH = 7.4) to remove non-adherent or loosely adhered bacteria. The bacteria were then stained with a solution of the FilmTracker^TM^ Live/Dead^®^ Biofilm Viability kit (Invitrogen, Life Technologies, Carlsbad, CA, USA). In this study, 1.5 µl of SYTO^®^9 and 1 µl of propidium iodide stain were added to 1 ml of PBS. After that, 150 µl of the staining solution was gently added to each substrate and plates were incubated for 15 minutes in the dark. Suspensions were then aspirated and the titanium substrates were transferred into a new well plate with ample PBS to fully immerse the sample. Samples were then visualized with a Nikon A1 confocal laser scanning microscope (CLSM) with a 40x water dipping lens. For the bacterial adhesion assay for 2 hours’ incubation, the surface coverage of bacteria under each field of view was determined by calculating the surface area of live and dead bacteria cells with ImageJ. For the early-stage biofilms formed after 2 days, the biomass under each field of view was determined by COMSTAT2 plugin (Lyngby, Denmark) in ImageJ. For each substrate type, three independent experiments were performed, and on each sampled surface, five equidistant evenly spaced fields (105.96 × 105.96 µm^2^) were selected.

### Scanning Electron Microscope Analysis

Samples were washed with PBS and fixed in 2% glutaraldehyde in 3 M Sorenson’s phosphate buffer overnight at 4 °C. Samples were transferred into a new plate and dehydrated through a series of ethanol solutions of 25% (v/v), 50%, 75%, and 100%, followed by critical point drying. The samples were sputter-coated with 24 nm platinum coating using a Cressington 328 UHR to improve the imaging quality in FEI Helios NanoLab 600 DualBeam system. The beam voltage and current were set to 5 kV and 0.34 nA, respectively. In addition, to reveal how the nanostructures interact with the cell membranes, focused Gallium ion beam (FIB) system (FEI Helios NanoLab 600 DualBeam™) was used to ion beam mill and examine the cross-section of bacteria and nanostructures. The beam voltage and current of the FIB system were set at 30 kV and 0.28 nA.

### Statistical Analysis

Data were represented by mean values with standard error. Student’s t-test assuming unequal variations was applied and *p < 0.05 was considered statistically significant in this study.

### Computational modelling for bacterial adhesion

The bacteria-material adhesion is governed by non-specific interaction energies that are dominated by attractive Lifshitz van der Waals (LW) forces and the electrostatic double layer (EL) interactions. These interaction energies are described in the classical DLVO (Derjaguin, Landau, Verwey, Overbeek) theory^62,63^. However, the DLVO theory ignores hydrogen and chemical bonds of the bacteria and material surfaces that also contribute to adhesion. Therefore, Van Oss *et al*. proposed the extended DLVO (XDLVO) theory by adding the short-range Lewis acid–base (AB) interactions to account for hydrogen bonding of bacteria and substrate surfaces^64,65^. The total interaction energy (ø) between a bacterium immersed in a liquid medium and the surface is given by:

1 $$\varnothing ={\varnothing }^{{\boldsymbol{LW}}}+{\varnothing }^{{\boldsymbol{EL}}}+{\varnothing }^{{\boldsymbol{AB}}}$$

It is well known that surface roughness can affect bacterial adhesion^7,19^. To gain a deeper understanding of the effects of surface roughness on bacterial adhesion, surface element integration (SEI) has been proposed^66^ and implemented to XDLVO^43^. The SEI technique converts the irreugular rough surface to a rough surface with hemi-spherical peaks and valleys, where the surface roughness and surface coverage remains the same. Usually, the peaks and valleys are assumed to be normally distributed^66^. Here, as the experimental measurements of surface roughness shows abrupt irregularities, a reconstruction of the three different surfaces was performed based on SEI. A normal distribution of mean surface rougness values for the polished and pocket-type surfaces was assummed since the peaks of roughness are distributed randomly on these surfaces, while a unifrom distribution was assumed for the spear-type surface which had a regular distribution of the peak roughness values.

The net interaction energy between the bacterium and surface can be calculated by integrating the net interaction energy per unit area (U) which is given by the following equation^43^:

2 $$U(D)={\int }_{0}^{2\pi }{\int }_{0}^{r}\varnothing (h)rdrd\theta $$

where *D* is the vertical distance between the bacteria and material surface, ø is the interaction energy as defined in Eq. (1), which depends on the seperation distance *h*. The detailed calculation of seperation distance has been explained by Ammar *et al*.^43^ *rdrdθ* represents the surface element of the cell surface. Discretisation of the bacterial cell surface is carried out in cylindrical coordinates. The simulation was carried out with an ionic strength of 10 mM for the given cell culture medium, and temperature of 298 K. Other parameters for simulation will come from the AFM measurement and wettability analysis in this study. Particularly, the zeta potential of titanium and the given bacteria were set to −30 mV and −8 mV^67^. An in-house C++ code was developed to implement the SEI enabled XDLVO theory to investigate bacterial adhesion.

## Electronic supplementary material

Supplementary information
