# Supplementary material for: Nanostructured titanium surfaces exhibit recalcitrance towards Staphylococcus epidermidis biofilm formation
Source: Sci Rep. 2018 Jan 18;8:1071. doi: 10.1038/s41598-018-19484-x (PMC5773551; doi:10.1038/s41598-018-19484-x)
Supplement: Supplementary file 1 — Supplementary information [file 41598_2018_19484_MOESM1_ESM.pdf]

Support Information

Nanostructured titanium surfaces exhibit recalcitrance towards *Staphylococcus epidermidis* biofilm formation

Yunyi Cao[1], Bo Su[2], Subash Chinnaraj[1], Saikat Jana[1], Leon Bowen[3], Sam Charlton[1], Pengfei Duan[1], Nicholas S. Jakubovics [4], and Jinju Chen[1]*

[1]School of Engineering, Newcastle University, Newcastle Upon Tyne, NE1 7RU, UK; [2]School of Oral and Dental Sciences, University of Bristol, Bristol, BS1 2LY, UK; [3]Department of Physics, Durham University, Durham, DH1 3LE, UK; [4]School of Dental Sciences, Newcastle University, Newcastle Upon Tyne, NE2 4BW, UK.

Corresponding Author

*E-mail: jinju.chen@ncl.ac.uk

Table S1. Chemical compositions of different titanium surfaces as revealed by XPS analysis.

Surfaces	***Carbon (At. %)***	***Oxygen (At. %)***	***Titanium (At. %)***
Polished	48.67 ± 4.18	39.38 ± 2.58	11.95 ± 1.35
Spear-type	26.71 ± 3.25	52.43 ± 3.56	20.86 ± 3.14
Pocket-type	25.24 ± 1.78	51.71 ± 2.45	23.05 ± 2.18

No differences were observed between the chemical compositions of the polished and hydrothermal-treated nanostructured titanium surfaces. However, there were small differences in the relative abundances of each element. After hydrothermal treatment, titanium was oxidized leading to higher Ti/O ratios.

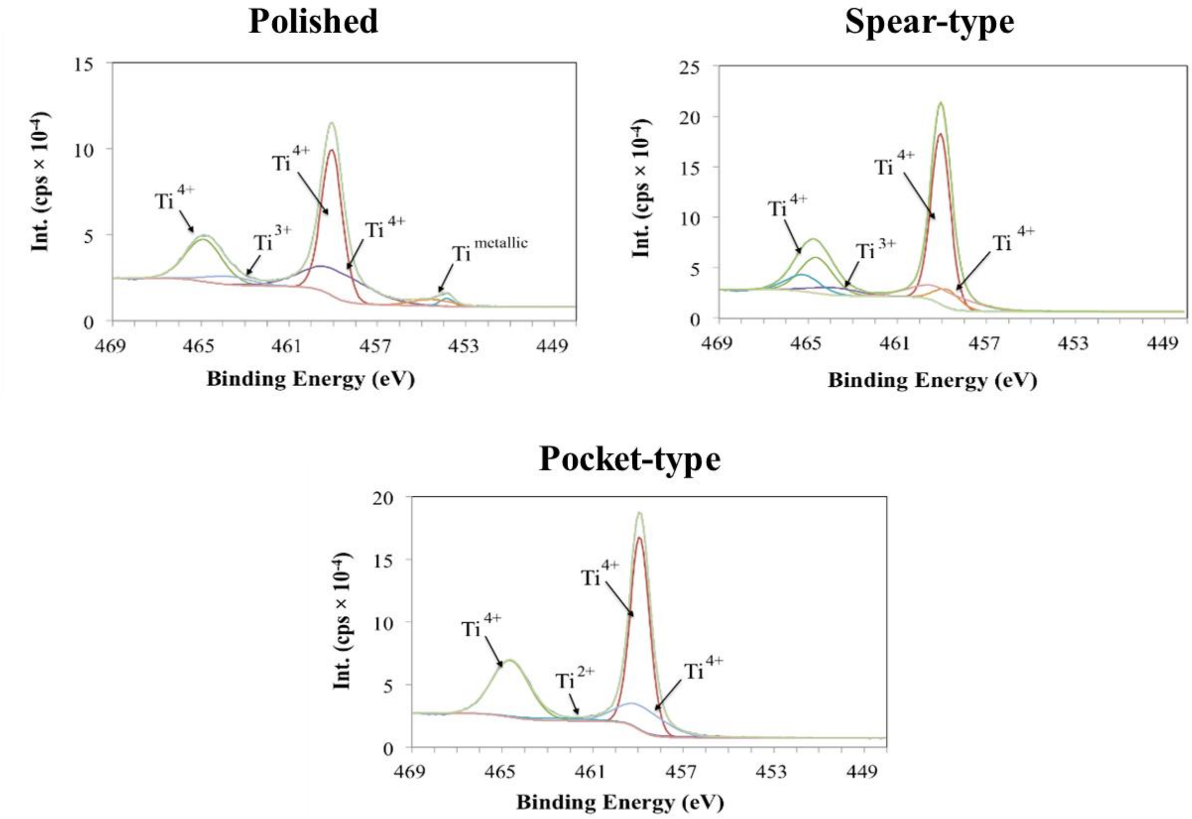

Figure S1. A high resolution XPS spectrum of the Ti 2p peak of titanium surfaces, for polished titanium, spear-type and pocket-type titanium, respectively.

High-resolution spectra of the Ti 2p core level of titanium surfaces were shown in Figure S1. The deconvolution of the peak unveils that there were several species that contain titanium at the surface. For polished titanium surface, it mainly contained metallic, TiO, and TiO_2. For spear-type titanium surface, it mainly contained Ti_2O_3, and TiO_2. For pocket-type titanium surface, it mainly contained TiO, and TiO_2. And both spear and pocket-type titanium surfaces did not contain metallic Ti, which means that they have been oxidized effectively on the surface layer. Also, all these three surfaces had the strong doublets (Ti 2p3/2 at around BE 458.8 eV and Ti 2p1/2 at around BE 464.5 eV), which were attributed to Ti^{4+} and indicated that titanium oxide were present mostly as TiO_2 on the surfaces of our samples.

Table S2. Contact angle measurement of DI water, diiodomethane and glycerol (degree) on different titanium surfaces.

Surfaces	*Contact angle of DI water (degree)*	*Contact angle of diiodomethane (degree)*	*Contact angle of glycerol (degree)*
Polished	82.80 ± 2.74	37.06 ± 0.62	69.27 ± 1.40
Spear-type	Spreading quickly	Spreading quickly	18.86 ± 0.86
Pocket-type	Spreading very quickly	Spreading very quickly	11.03 ± 1.21

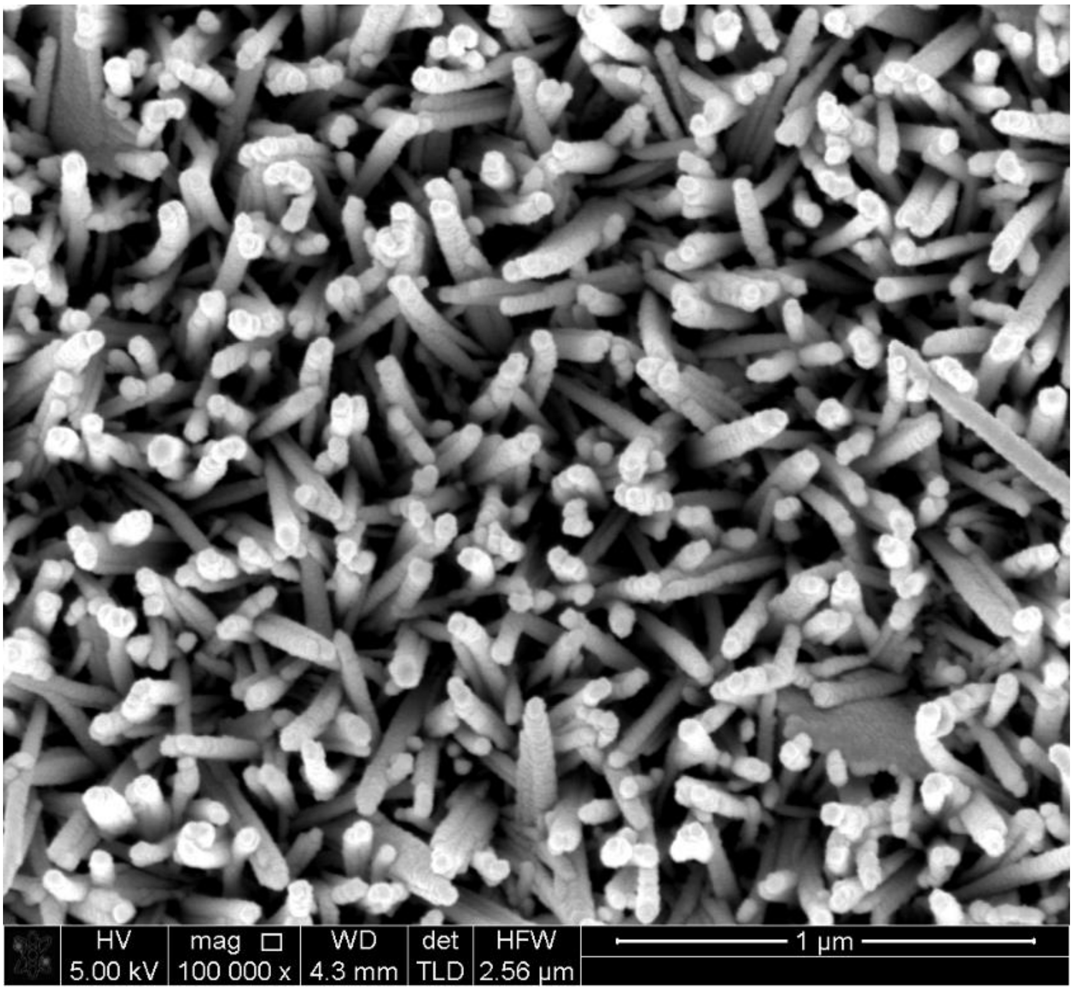

Figure S2. A high resolution SEM image of spear-type titanium surface with the magnification of 100,000×. Initially, one spear was randomly chosen, and the distance between the spears (the nearest spears that around the chosen one) were manually measured by ImageJ. Totally 5 random nano-spears were chosen and the average distance between the nano-spears was measured to be 191.09 ± 120 nm.

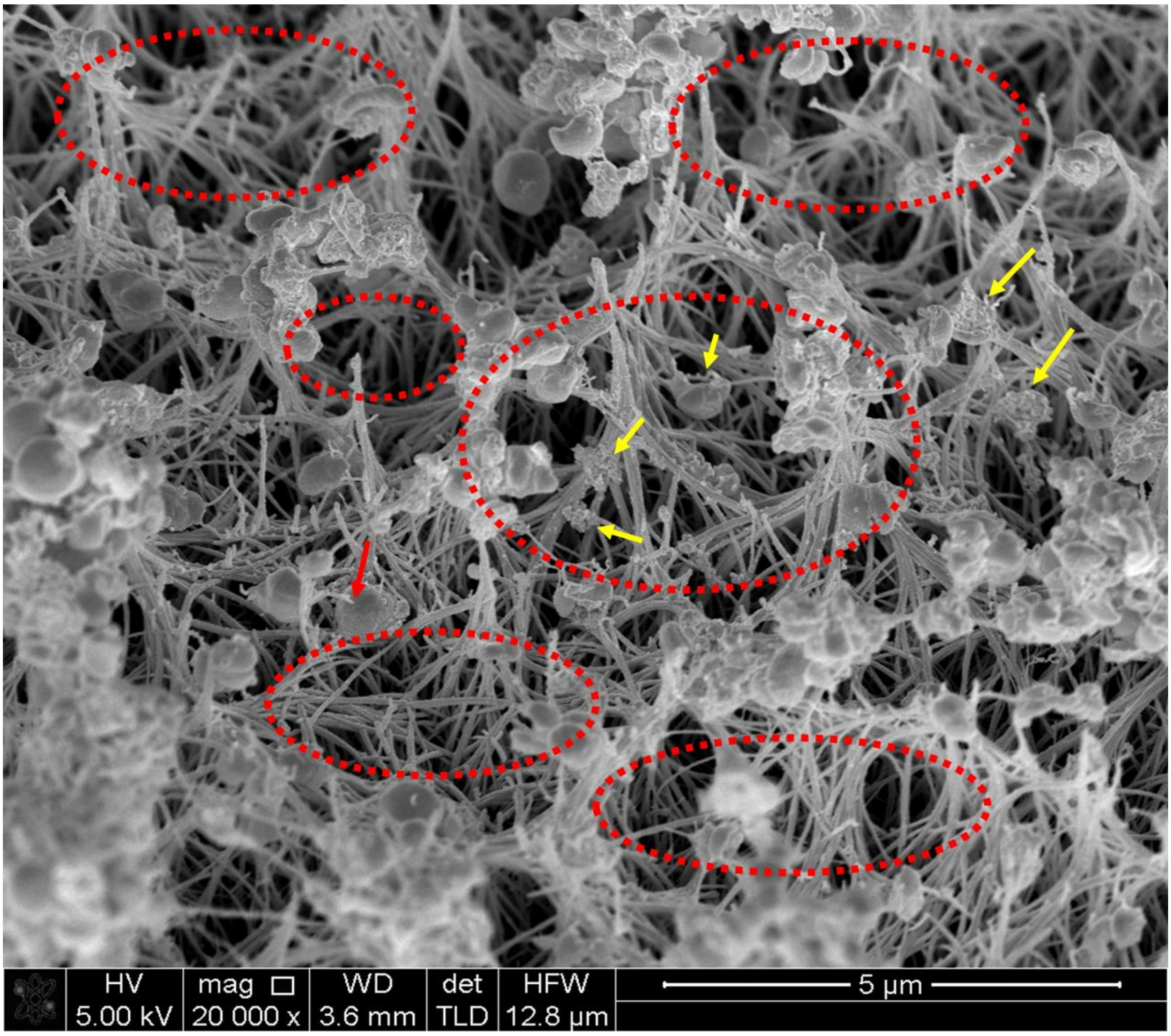

Figure S3. The typical SEM image with 40° tilt of *S. epidermidis* biofilms grown on the pocket-type titanium surface over a period of 6 days, taken at the magnification of 20000 ×. Red arrows indicated the direct penetration from the spears. Yellow arrows indicated the decay of dead cells inside the pockets (red dash circles).

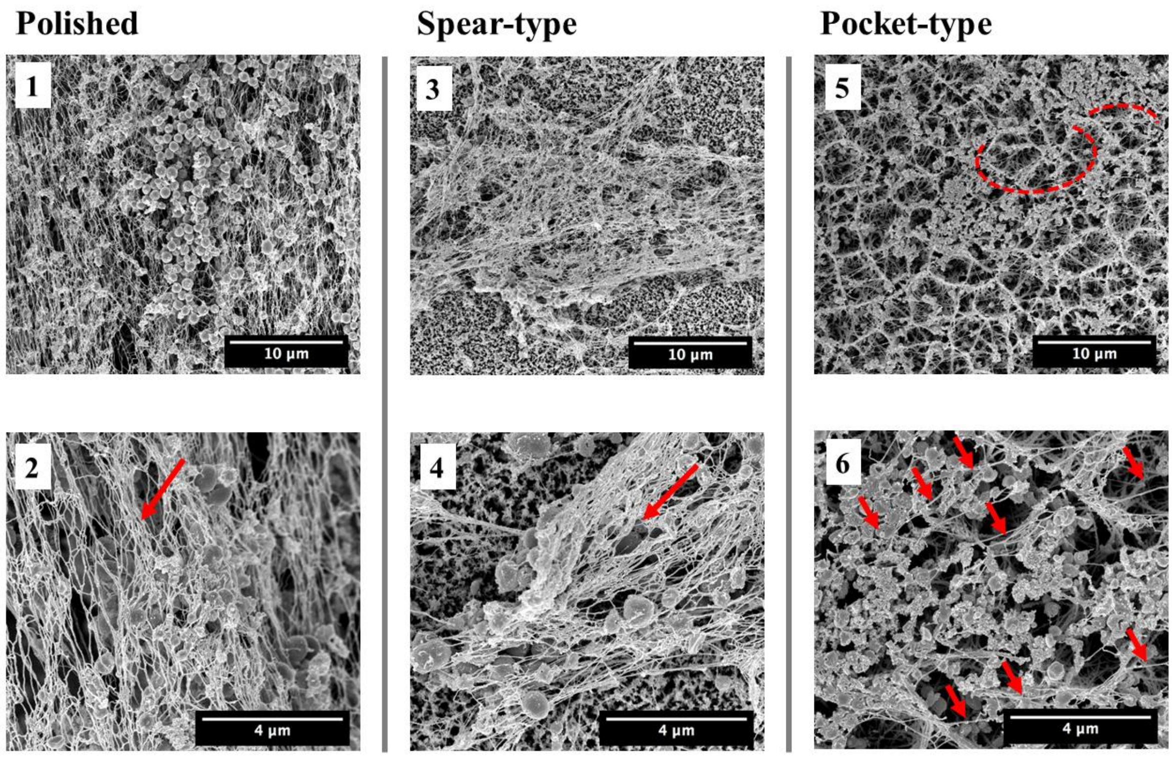

Figure S4. *S. epidermidis* biofilms grown on different surfaces over a period of 9 days. The images (1, 3, and 5) were taken at the magnification of 8000 ×; Images (2, 4, and 6) were higher magnification (25000 ×) of the biofilms. More mature and dense biofilms were found on the polished and the spear-type titanium surfaces, with a large amount of EPS visible (red arrows). By contrast, the pocket-type surfaces continued to exhibit small bacterial clusters and tended to wrap (red dash lines) around at the nano-spears that form the pockets with little EPS observed (red arrows).
